# Lenalidomide in Diffuse Large B-Cell Lymphomas

**DOI:** 10.1155/2012/498342

**Published:** 2012-06-27

**Authors:** Annalisa Chiappella, Umberto Vitolo

**Affiliations:** Hematology 2, San Giovanni Battista Hospital and University, c.so Bramante 88, 10126 Torino, Italy

## Abstract

Diffuse Large B-cell Lymphomas (DLBCL) are the most frequent Non-Hodgkin Lymphomas (NHL). The addition of Rituximab to the standard chemotherapy CHOP improved the outcome in this patients, but so far 40% of patients experienced relapse or progressive disease. Lenalidomide, an immunomodulatory agent, had direct tumoricidal and antiangiogenetic actions on tumor cells and was able to modulate tumor-cell microenvironment, with the restoration of impaired T-cell activity and the formation of immuno-synapsis. Based on these actions, lenalidomide represented an active drug on aggressive relapsed NHL. In this review, the most relevant clinical trials for the use of lenalidomide in DLBCL were reported. Monotherapy with lenalidomide showed an activity in term of overall response rate, with acceptable hematological and extrahematological toxicities in relapsed/refractory aggressive NHL. The role of lenalidomide as salvage therapy in both cell of origin patterns in DLBCL (germinal center B-cell/activated B-cell) was reported in preliminary data. Preliminary data regarding the role of lenalidomide in addition to chemoimmunotherapy (R-CHOP) in first line clinical trials were discussed; data of safety, feasibility and efficacy were promising.

## 1. Introduction

**Figure 1:**
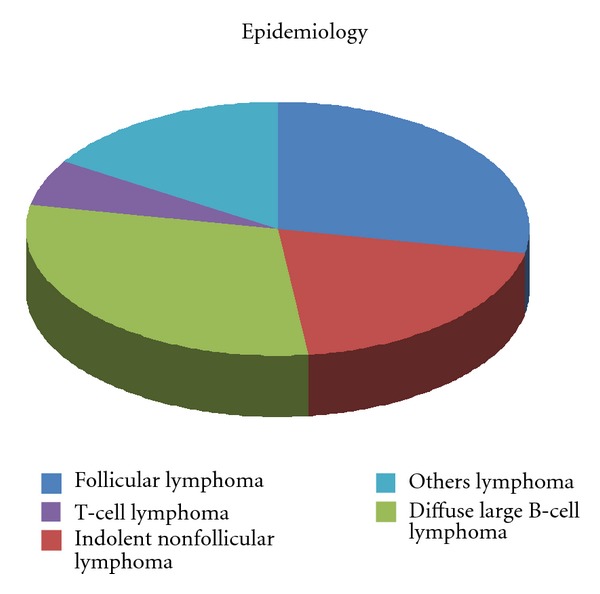
Incidence of Non-Hodgkin Lymphoma.

Diffuse large B-cell lymphoma (DLBCL) represents roughly 40% of all non-Hodgkin lymphoma (NHL) (Figure 1), with a rate of incidence in continuous increase and median age at diagnosis of 55–60 years [1, 2].

The addition of monoclonal antibody anti-CD20 rituximab to standard chemotherapy CHOP (cyclophosphamide, doxorubicin, vincristine, and prednisone) has improved the outcome compared to CHOP alone in untreated DLBCL elderly patients with a complete remission (CR) rate of 75% versus 63% [3]; the advantage of R-CHOP versus CHOP was maintained at a median followup of ten years; Overall Survival (OS) of 43.5% versus 27.6%; Progression Free Survival (PFS) of 36.5% versus 20.1%, respectively [4]. Also in combination with dose-dense chemotherapy CHOP14 the rituximab showed promising results in elderly untreated DLBCL [5]. In order to ameliorate prognosis, with the support of granulocyte colony-stimulating factor (G-CSF), dose-dense chemotherapy CHOP14, administered every 14 days, with or without rituximab, was tested in elderly DLBCL at diagnosis; RICOVER-60 trial showed the superiority of 6 courses of R-CHOP14 compared to CHOP14, with 3-year event-free survival 66.5% versus 47.2% and 3-year overall survival 78.1% versus 67.7%, respectively [5]. In young patients affected by poor prognosis DLBCL at diagnosis, rituximab plus dose-dense chemotherapy plus high-dose chemotherapy and autologous stem cell transplant were tested, with promising results (4-year PFS 73% and 4-year OS 80%) [6].

Despite the improvement of outcome with chemoimmunotherapy, rituximab plus dose-dense chemotherapy or high-dose chemotherapy plus autologous stem cell transplant, 30–40% of patients relapsed after first line treatment, and the rate of second CR in patients pretreated with rituximab chemotherapy is lower than 30% [7].

It will be mandatory to obtain a better CR in first line DLBCL and, in relapsed or refractory patients, to overcome chemorefractoriness; the introduction of novel drugs represents a chance to obtain these goals.

In the landscape of novel drugs, immunomodulating drugs (IMiDs) represent now a real opportunity to ameliorate prognosis in DLBCL.

## 2. Lenalidomide: Mechanism of Action and Rationale

**Figure 2:**
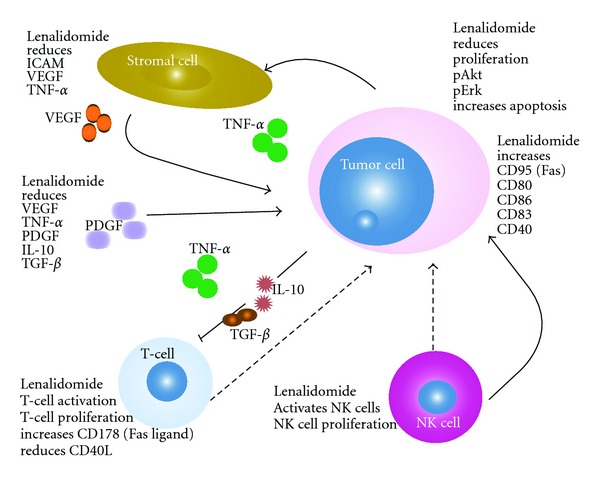
Action of lenalidomide.

Lenalidomide, CC-5013, is an immunomodulatory agent with multiple mechanisms of action and it is an active agent on aggressive NHL, blocking tumor growth and survival with direct tumoricidal and immunomodulatory actions. This drug has both antiproliferative and antiangiogenic activities. Lenalidomide's activity is based on modulation of tumor-cell microenvironment and on stimulating the activity of effector cells, such as cytotoxic T and natural killer cells. Lenalidomide enhanced T-cell and NK-cell effector function to eliminate tumor B cells and it had a role in the restoration of impaired T-cell activity and formation of immunologic synapses [8] (see Figure 2).

Lenalidomide was initially introduced in the treatment of multiple myeloma and only in a second time was tested in lymphoma cell lines. In animal models of lymphoma, IMiDs and especially lenalidomide demonstrated a synergic action with rituximab; the addition of lenalidomide to rituximab increased median survival in mice from 45 days to 58 days compared to rituximab alone [9]. Another study demonstrated that IMiDs increased the recruitment of natural killer cells to subcutaneous lymphoma sites in mice with the stimulation of dendritic cells and modification of the cytokine microenvironment; lenalidomide, in association with rituximab, increased antibody-dependent cellular cytotoxicity [10].

## 3. Lenalidomide: Clinical Experience in Relapsed/Refractory DLBCL

On the basis of the *invivo* activity of IMiDs, Wiernik et al. conducted a phase II multicenter trial to evaluate safety and efficacy of lenalidomide monotherapy in relapsed/refractory aggressive lymphomas patients. Forty-nine patients were enrolled to receive oral lenalidomide 25 mg once daily on days 1 to 21, every 28 days, for 52 weeks, until disease progression or intolerance. Median age was 65 years, 53% of patients had DLBCL, and all of them received at least four prior therapeutic regimens; 92% of patients had received prior rituximab and 29% of them had been previously transplanted. The overall response rate (ORR) was 35% for all histology and 19% for DLBCL. The estimated median duration of response was 6.2 months (range: 0 to 12.8 months) and median PFS was 4 months (range: 0 to 14.5 months). Regarding safety, the most common grade 4 adverse events were neutropenia (8.2%) and thrombocytopenia (8.2%); the most common grade 3 adverse events were neutropenia (24.5%), leukopenia (14.3%), thrombocytopenia (12.2%), and thrombocytopenia in 8.2%, resolved with dose reduction. The results showed that lenalidomide monotherapy is active in relapsed or refractory aggressive NHL, with manageable side effects [11]. The same schedule of lenalidomide was tested by Witzig in the NHL-003 international phase II trial for relapsed or refractory aggressive B-cell non-Hodgkin's lymphoma. Two hundred and seventeen patients were enrolled and 108 had DLBCL. In all histologic subgroups, ORR was 35% with CR 13%, partial remission (PR) 22%, and stable disease 21%; ORR for DLBCL was 28%. Moreover, ORR was 37% for patients who underwent prior stem cell transplantation and 33% for rituximab refractory ones. Median PFS for all 217 patients was 3.7 months; for 77 responders, the median response duration lasted 10.6 months. Despite the fact that patients were heavily pretreated, lenalidomide was well tolerated. The administered median daily dose of lenalidomide was 25 mg (range 7.1–25 mg) and 117 patients (53.9%) required at least one dose reduction or interruption due to neutropenia in 56% and thrombocytopenia in 31%. Grade 3 or 4 adverse events included neutropenia in 41%, with only 2% of febrile neutropenia, thrombocytopenia in 19%, and anemia in 9.2%. Discontinuation from study treatment occurred in 49 patients (23%). Extrahematological toxicities included tumor flares in 7 patients, 4 (1.8%) with grade 1 or 2 and 3 with grade 3, gastrointestinal events in 61.3%, rash in 18% and fatigue in 28%. Granulocyte colony stimulating factors (GCSF) were administered to 54 patients (25%) during the study [12].

In the last decades, an important role to predict the outcome in DLBCL was represented by gene expression profiling and by pattern of origin, germinal center B-cell and nongerminal, or activated B-cell-like. The outcome of the two subgroups seems to be different, with a worst outcome in activated B-cell lymphoma. In a recent study, Hernandez-Ilizaliturri retrospectively analyzed clinical outcomes of 40 patients with relapsed/refractory DLBCL, 23 germinal center, and 17 nongerminal center, treated with salvage lenalidomide as single agent. Median age was 66 years and median number of prior treatments, including rituximab plus chemotherapy, was four. Germinal center and nongerminal center B-cell subgroups were similar in terms of stage, international prognostic index score, prior number of treatments, and rituximab resistance. Results demonstrate a different antitumor responsiveness in the two biological subgroups: ORR for nongerminal (activated B cell) versus germinal B cell was 52.9% versus 8.7% (*P* = 0.006), CR was 23.5% versus 4.3%, and median PFS was 6.2 versus 1.7 months (*P* = 0.004), respectively; no difference was observed regarding overall survival. The advantage in treatment with lenalidomide seems to be increased in nongerminal subtype compared to germinal subgroup; this hypothesis should be based on the different expression of NF-*κ*B, targeted by IMiDs, in the two subgroups [13]. A large international trial (NCT01197560) to compare lenalidomide to investigator's choice is still ongoing; in this clinical study patients will be risk-stratified according to germinal center/nongerminal center B-cell-like in order to identify the subgroup that benefit from lenalidomide treatment.

The efficacy of lenalidomide in monotherapy and the well-tolerated profile, supported the rationale for investigating in a phase II trial the efficacy and safety of the combination of lenalidomide and rituximab in pretreated elderly patients with DLBCL. Lenalidomide was administered at the dose of 20 mg/daily from day 1 to 21 every 28 days, for four courses, in combination with 375 mg/sqm rituximab on day 1 and day 21 every 28 days for four cycles. Responsive patients (CR, PR, or stable disease) were given lenalidomide maintenance therapy at the same schedule for an additional eight months. Twenty-three elderly DLCL patients at median age 74.2 years, heavily pretreated with a median of three prior therapies (range, 2 to 8), were enrolled and treated with rituximab plus lenalidomide. After the induction phase, the ORR was 35%, with 7 patients achieving a CR, one patient a PR, 2 stable disease, and 13 progressive disease. Ten patients were eligible for lenalidomide maintenance. At the end of the entire treatment regimen, CR was 35%. At a median follow-up of 16 months, the estimated 1-year-disease-free-survival was 34.8% and the 18-month OS rate for the whole study population was 55.1%. Therapy was feasible with low rate of grade 3 or 4 toxicities [14]. 

Ivanov described a case report of a 65-year-old DLBCL patient relapsed after four lines of chemotherapy that included high-dose chemotherapy plus autologous stem cell transplantation. Lenalidomide was administered at the dose of 15 mg per day for 21 days every 28 days in association with 375 mg/sqm rituximab on day one and 40 mg oral dexamethasone on day, one and four. Seven courses were administered, obtaining CR; patient was in remission at 20 months after the end of treatment [15].

Lenalidomide as single agent was also tested on transformed lymphoma, such as transformed follicular lymphoma and transformed chronic lymphocytic leukemia/small lymphocytic lymphoma. Thirty-three patients were treated with 25 mg lenalidomide for 21 days every 28 days; ORR was 46%, with a median response duration of 12.8 months. Median PFS was 5.4 months. Among 23 patents with transformed follicular lymphoma, ORR was 57%, with 26% of CR; among 7 patients with transformed chronic lymphocytic leukemia/small lymphocytic lymphoma, ORR was 0 and none reached CR. Neutropenia grade 3 and 4 were observed in 33% and 15%, respectively, grades 3-4 thrombocytopenia in 5% and grade 3 pneumonia in 3% [16].

## 4. Lenalidomide: Clinical Experience and New Options in Untreated DLBCL

The promising results of lenalidomide in relapsed/refractory DLBCL setting, encouraged the development of trial with this drug in first line treatment.

Considering that safety and efficacy of lenalidomide in combination with standard immunochemotherapy was unknown, some phase I trials were drawn with the aim to define the Maximum Tolerated Dose (MTD) and the Dose-Limiting Toxicities (DLT) of lenalidomide in addition to standard therapy rituximab-CHOP. Nowakowski conducted a phase I/II study to define MTD and efficacy of lenalidomide administered on days 1–10 with standard R-CHOP chemotherapy (R2CHOP) in 24 newly diagnosed DLBCL and grade 3 follicular lymphomas; median age was 65 years (range 35–82) and 54% of patients were at low-intermediate IPI score. Lenalidomide dose escalation levels were 15 mg, 20 mg, and 25 mg. All patients received 6 mg pegfilgrastim on day 2 and aspirin prophylaxis. Dose-limiting toxicity (DLT) was defined as any grade 3 or higher nonhematological toxicity or a hematological toxicity resulting in a delay of the next cycle of chemotherapy. In the phase I, three patients received 15 mg, 3 patients 20 mg, and 18 patients 25 mg of lenalidomide; no DLT was found and 25 mg days 1–10 was the recommended dose for phase II. The incidence of grade 4 neutropenia or thrombocytopenia was 67% and 21%, respectively; no toxic deaths were recorded. ORR was 100% with CR in 77% of patients [17].

A similar schedule was tested by the GELA group; 27 patients affected by follicular lymphoma in 18, DLBCL in 4, mantle-cell lymphoma in 3, and indolent lymphoma in 2 were treated with oral lenalidomide on days 1–14 in association with R-CHOP given every 3 weeks for 6 cycles. Lenalidomide dose was increased from 5 mg to 25 mg (5 mg per dose level), using a 3 + 3 escalation design. Pegfilgrastim was administered on day 4 and oral aspirin prophylaxis (100 mg) was given daily during the treatment. Maximum-tolerated dose was determined by the number of DLT during the first 2 cycles. DLT was defined as grade 3 or more nonhematological toxicity, grade 3 hematological toxicity lasting more than 7 days, or grade 4 hematological toxicity lasting more than 3 days. Results showed that 25 mg was considered as the recommended dose. Most frequent adverse event was grade 3-4 neutropenia in 59%, including 7% of febrile neutropenia, and grade 3-4 thrombocytopenia in 30%. No grade 3-4 neurological toxicities were observed. One patient had pulmonary embolism of moderate severity and one patient has a deep vein thrombosis. Lenalidomide was stopped in five patients due to toxicity according to protocol defined criteria [18].

**Figure 3:**
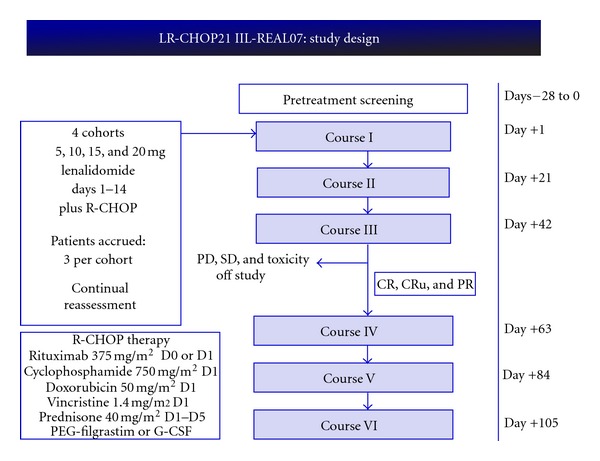
Phase I/II trial of Italian Lymphoma Foundation for elderly untreated DLBCL.

The Italian Lymphoma Foundation conducted a phase I-II trial to test the combination of lenalidomide plus R-CHOP (REAL07) in newly diagnosed DLBCL elderly patients not eligible to high-dose chemotherapy plus stem cell transplant. The treatment scheme is described in Figure 3. At the end of phase I part of the trial, MTD for lenalidomide in association with R-CHOP21 resulted as 15 mg days 1–15. The association was well tolerated, with grade 3-4 thrombocytopenia and neutropenia as expected and low rate of neurological toxicities [19].

Several studies with lenalidomide in association with standard treatment are ongoing in first line DLBCL patients. One of these, the REMARC study, is designed to demonstrate if a maintenance with lenalidomide after first line conventional chemoimmunotherapy may improve PFS compared to observation only.

## 5. Conclusions

The introduction of IMiDs in the treatment of DLBCL represented an improvement in the outcome of this patients. Lenalidomide represents a manageable drug, with good results in relapsed or refractory DLBCL patients heavily pretreated. The role of lenalidomide in association to standard chemoimmunotherapy RCHOP in first line is under investigation, with promising results in term of feasibility, toxicity and with promising results in term of response. The activity of lenalidomide in histological subtypes at poor outcome, like in activated B-cell DLBCL, may be demonstrated in prospective trials.

Lenalidomide should be considered as conventional treatment in relapsed/refractory setting of patients in monotherapy or in association with rituximab and/or steroid. Ongoing trials should clarify the feasibility of lenalidomide in association with other drugs, such as with platinum containing regimens (oxaliplatinum-cytarabine or carboplatinum-idarubicine-etoposide) or with mTOR inhibitors (everolimus and temsirolimus) or with bendamustine or with monoclonal antibodies (GA-101). The role of maintenance of lenalidomide after first line chemoimmunotherapy should be established by ongoing trials.
